# Orbital Sarcoidosis: A Brief Guide to an Insidious Disease

**DOI:** 10.7759/cureus.91990

**Published:** 2025-09-10

**Authors:** Donika I Vezirska, Vladimir S Prandzhev

**Affiliations:** 1 Department of Neurosurgery, Military Medical Academy, Sofia, BGR

**Keywords:** idiopathic orbital inflammation, orbital lesions, orbital pseudotumor, orbital sarcoidosis, sarcoidosis

## Abstract

Sarcoidosis is a rare systemic disease that can affect any organ. Orbital manifestations constitute an uncommon extrapulmonary form of sarcoidosis. The symptoms and imaging findings can mimic those of other pathologies, such as orbital pseudotumor lesions, thus delaying clinical diagnosis and adequate treatment. We systematically reviewed the existing literature on the topic, published in PubMed and Google Scholar, up to August 2025, regarding the etiology, clinical presentation of the disease, diagnostic methods, and management. Search words included “orbital sarcoidosis,” “sarcoidosis/”orbital,” and variations of these terms. Only English-language articles were selected. All broader articles that dealt with ocular sarcoidosis or neurosarcoidosis were excluded. The literature review is based on 18 articles (case reports, case studies, and original articles). It presents the current data for orbital sarcoidosis in five relevant sections: a brief review of the relevant anatomy of the orbit, presentation, diagnostic methods and evaluation, management of the disease, differential diagnosis, and related diseases. Sarcoidosis remains a challenging pathology to diagnose and manage. Its orbital manifestation is an insidious imitator that can undermine timely treatment and lead treating medical professionals to a false diagnosis. Along with the nonspecific clinical presentation, orbital sarcoidosis can be a peculiar disease that goes unnoticed for a long period.

## Introduction and background

Sarcoidosis is a rare chronic multisystemic disease that is histopathologically characterized by non-caseating granuloma formation. It is more commonly associated with the female sex and European origin, and affects patients between the second and fourth decades of life. In more than 90% of the cases, the lungs are affected by the disease, while up to half of the cases in general have extrapulmonary manifestations, the most common ocular one being uveitis. Although ocular sarcoidosis is well-described, isolated orbital involvement is rare, underrecognized, and often misdiagnosed.

Orbital sarcoidosis is a very uncommon manifestation that can be either discrete or diffuse. It can affect the soft tissues of the orbit, the lacrimal gland, the optic nerve, and the extraocular muscles. Eyelids may also be involved, as well as the lacrimal sac and the nasolacrimal duct. The lesion is mostly unilateral [1]. This manifestation can be the first sign of sarcoidosis in approximately 65% of patients with sarcoid-like granulomatous orbitopathy, although statistical data are scarce due to the rarity of the disease [2].

Symptoms of orbital sarcoidosis are often nonspecific, and even an experienced physician can be misguided. Oftentimes, the first sign of the disease is a slow-growing palpable orbital mass. Other symptoms include swelling and different displacements of the globe, as well as ptosis and proptosis. Redness, pain, and visual field deficits may also occur. Differential diagnosis may include diseases presenting with idiopathic orbital inflammation, tuberculosis, cellulitis, and lymphoproliferative disorder [3].

Differentiating the tissues affected by orbital sarcoidosis is important for the precise localization of the lesion and subsequent management, especially if surgical debulking is planned. The orbit is an anatomical space that houses the globe, extraocular muscles, nerves, blood vessels, lacrimal apparatus, and adipose tissue. Orbital sarcoidosis is a manifestation that most commonly affects the lacrimal apparatus. Furthermore, it shows a predilection for the anterior inferior quadrants of the orbit [4].

This review will discuss the anatomy, presentation, diagnosis, and management of orbital sarcoidosis, based on a review of 18 cases from the literature. It aims to describe a simple guide and management strategy regarding orbital sarcoidosis.

## Review

Methodology

The narrative review was conducted using online databases, such as PubMed and Google Scholar. Search words included “orbital sarcoidosis,” “sarcoidosis/”orbital,” and variations of these terms. Only English-language articles were selected. Search results were not limited to a specific period; however, most of the articles were published before 2017. Articles containing data regarding the etiology, clinical presentation of the disease, diagnostic methods, and its management were included. All broader articles that dealt with ocular sarcoidosis or neurosarcoidosis were excluded.

Results

This narrative literature review is based on 18 articles (case reports, case studies, and original articles). It presents the current data for orbital sarcoidosis in five relevant sections: a brief review of the relevant anatomy of the orbit, presentation, diagnostic methods and evaluation, management of the disease, differential diagnosis, and related diseases.

Discussion

Orbital sarcoidosis is uncommon. It can be mistaken for isolated orbital granulomatous disease, especially when the lack of systemic disease is overlooked. This is an issue for the research material on the topic because a significant number of the publications don’t include systemic disease as a criterion for the inclusion of cases. Isolated orbital granulomatous disease affects mostly men in the fourth decade of life and involves the lacrimal gland in over 50% of cases [2,4].

Brief Review of the Relevant Anatomy of the Orbit

The orbit is a bony pyramid-like structure with a floor, a roof, and medial and lateral walls that present the sides of a 45-degree angle [5]. Due to the lateral rotation of the base of this pyramid, also known as the orbital entrance, the lateral orbital rim is situated approximately at the equator of the globe, resulting in the relatively lateral exposure of the globe. The extraocular muscles include the superior, inferior, medial, and lateral rectus muscles, and the superior and inferior oblique muscles. The rectus muscles originate from the annulus of Zinn, and together with the intramuscular septa, separate the intraconal from the extraconal space. The extraocular muscles are supplied by the oculomotor nerve (III) except the lateral rectus, which is supplied by the abducens nerve (VI), and the superior oblique, which is supplied by the trochlear nerve (IV). The ophthalmic artery serves as the main arterial supply of the orbit with contributions from the maxillary and middle meningeal arteries, branches of the external carotid artery. There are important anastomoses between the internal and external carotid circulations.

The globe and the other orbital tissue structures are surrounded by orbital fat which provides cushioning to the globe and aids movement. The orbit is lined by the periorbita which is a continuous fibrous membrane. It forms dense condensations at different margins and suture lines, otherwise, it is loosely attached and allows for a relatively bloodless entry into the subperiosteal space.

Presentation

A subtle start and absence of pain are characteristic of an atypical orbital inflammation such as sarcoidosis; an acute onset is very uncommon [6]. Leukocytosis and acute fever are also uncommon. The patient is most commonly in good condition and presents with a palpable orbital mass and eyelid swelling [7]. If the lacrimal gland is involved, symptoms can also include ptosis/proptosis and globe displacement. The history of the disease should be extensively examined to rule out other systemic autoinflammatory pathology such as Ig-G4-related disease and sclerosing orbital inflammation. Other symptoms may include redness, pain, vision loss, tearing, blurring, and diplopia, although they are very rare [8].

Osseous involvement is very uncommon and presents with rapid onset, no afferent pupillary defect (APD), and severe vision loss, as seen in one case report. Presumably, this happens because the process develops in the posterior orbital space [9]. Similar symptoms in a second case report were observed and multifocal bony disease was found. Sarcoidosis was confirmed with pulmonary CT [10]. This advancement of the disease is thought to correlate with a worse prognosis.

Diagnostic Methods and Evaluation

Some studies show the use of angiotensin-converting enzyme (ACE) levels testing when in doubt for sarcoidosis, as it is produced by the epithelial cells surrounding the inflammatory granulomas associated with it. However, the percentage of patients with elevated ACE levels in case study groups varies greatly (between 60 and 90%). Moreover, there are several non-sarcoid conditions, including diabetes mellitus, osteoarthritis, and hyperthyroidism that may cause this phenomenon. This makes ACE, be it in serum or in the cerebrospinal fluid (CSF), an inconsistent and unreliable laboratory indicator.

Biopsy is the gold standard method that allows for the histological examination of the process but it is common that the histological examination is not definitive. Still, it is not clear what exactly makes the diagnosis difficult but it is worth noting that the pathology is rare and it has few characteristic features that can serve as reference points [8]. However, the finding of a chronic non-caseating granulomatous process should lead to further testing and imaging diagnostics. An electrocardiogram is also reported to be useful for screening patients with suspected sarcoidosis because of possible cardiac involvement [11].

Gallium scintigraphy can be used to evaluate the orbital status in patients with normal chest radiographs [1]. Attention should be paid to the characteristic panda eye sign, indicating bilateral symmetrical involvement of the lacrimal and parotid glands, thus significantly narrowing the spectrum of possible diagnoses.

CT and MRI of the head complement each other when complex differentiation between pathologies are made. CT imaging is superior for imaging bony structures and demonstrating contrast between orbital fat, extraocular muscles, paranasal sinuses, and areas of inflammation. MRI is superior in terms of soft tissue structures as well as imaging of the orbital apex, cavernous sinus, and superior orbital fissure. The inflammatory infiltrate usually has a low signal on T2-weighted images (T2WI), whereas a high signal means malignant lesions such as metastatic disease or lymphoproliferative disorders [12]. The affliction known as orbital pseudotumor may present with a discrete retrobulbar mass or diffuse infiltration of the orbital contents. This is characteristic of the most common form of orbital sarcoidosis [1,12].

Chest CT is superior to X-ray for imaging diagnostics of systemic sarcoidosis because of the fewer number of false-negative findings it yields and its higher sensitivity [4,13].

Management

Three main types of treatment can be undertaken: conservative treatment, radiotherapy, and surgical debulking. Although different studies suggest that the condition can enter remission spontaneously, the data to recommend merely observation is insufficient [4]. 

The most common approach to the management of orbital sarcoidosis, regardless of the exact localization, is conservative treatment with oral steroids. Depending on the status of the systemic disease, a short prednisolone course may be started. Doses start from 1 mg/kg and may be reduced over over months [13-15]. In case of therapy failure or steroid resistance, methotrexate is indicated. Several case reports show the successful combination of prednisone and methotrexate [9,16]. Anti-tumor necrosis factor (TNF) alpha therapy, most notably infliximab, is also considered as a potential therapy option [15].

For localized lesions, a periorbital injection of steroids (triamcinolone acetonide) may be indicated [4]. This is effective with patients who show systemic intolerance towards steroids. Radiation therapy is another method that is used in steroid-refractory and chronic cases. However, surgical decompression is more common, especially when the lesion is well-circumscribed and located more anteriorly [8,15].

Differential Diagnosis and Associated Diseases

Diseases that should be taken into account when discussing the possibility of orbital sarcoidosis include granulomatosis with polyangiitis, thyroid eye disease, sickle cell disease, cocaine or bisphosphonate use, IgG4-related disease, and sclerosing orbital inflammation [1,17,18]. Orbital sarcoidosis should always be considered in cases of unexplained orbital masses, especially when systemic symptoms are present. We have reviewed the differential diagnosis of orbital sarcoidosis in Table 1.

**Table 1:** Differential diagnoses according to key symptoms in the history of the disease and specific findings in terms of physical/imaging examinations and laboratory tests [1-18]

Symptoms	Disease	Physical examination findings	Imaging examination findings	Laboratory testing
Glomerulonephritis and sinusitis	granulomatosis with polyangiitis	proptosis, extraocular dysmotility, nasolacrimal duct obstruction, ocular inflammation	bony erosion of the sinuses, fibrosclerotic inflammation	positive antineutrophil cytoplasmic antibodies (ANCA)
Anxiety, palpitations, heat/cold intolerance, and weight loss	thyroid eye disease	usually bilateral, eyelid retraction, extraocular muscles often restricted and weak, dull retroocular ache (exacerbated by eye movements)	enlargement and stiffness of the extraocular muscle bodies, with tendon sparing, with the medial and inferior rectus muscles being most involved, increase in orbital fat	elevated thyroid-related antibodies, abnormal thyroid function tests - most commonly hyperthyroidism
Orbital compartment syndrome, extraocular dysmotility, chemosis	sickle cell disease	pain, proptosis, compressive optic neuropathy, children or young adults	hemorrhagic and exudative subperiosteal collections along the lateral orbital wall, edema due to sphenoid wing infarction	complete blood count, hemoglobin electrophoresis, high-performance liquid chromatography
Cocaine-related sinoorbitopathy	cocaine use	chronic sinusitis	loss of the nasal septum, bony erosion of the sinuses, erosion of portions of the medial orbital wall	specific toxicology screen for cocaine and its metabolites
Acute onset of orbital inflammation	bisphosphonate use	treatment for osteoporosis, osteolytic bone tumors, skeletal metastatic disease, Paget’s disease	diffuse involvement	-
Retroperitoneal fibrosis, pancreatitis, cholangitis, hydronephrosis	IgG4-related disease and sclerosing orbital inflammation	asthma, allergy	enlargement of the infraorbital nerve/canal	elevated serum levels of IgG4

Limitations

Currently, a major limitation of this review is the complexity of differentiating orbital from ocular sarcoidosis. This is a point to consider when reviewing the literature on the matter and many articles that were eliminated during the literature search did not notice the significant difference between the two variations. The other major limitation is the small number of studies which does not allow for a meta-analysis that may enhance the credibility of the study. The numbers concerning the incidence of symptoms vary greatly which may be the result of the limited number of cases.

## Conclusions

Orbital sarcoidosis remains a clinical challenge even for experienced physicians. The growing incidence of idiopathic inflammatory disease necessitates the need for more research in the field of systemic disease in general. However, there were a limited number of thoroughly described cases of this specific variety of sarcoidosis. This review provides a simple guide to the diagnostic process and the current trends in the clinical management of orbital sarcoidosis. More research in this area is needed, as there is a marked lack of prospective studies and a limited consensus on the management of this manifestation of sarcoidosis. Our study may aid in the need for standardized diagnostic criteria as it supplies a clear guide to orbital sarcoidosis in the context of precise anatomical differentiation.

## References

[1] Ferreira TA, Saraiva P, Genders SW, Buchem MV, Luyten GP, Beenakker JW (2018). CT and MR imaging of orbital inflammation. Neuroradiology.

[2] Vahdani K, Rose GE (2021). Sarcoid-like granulomatous orbitopathy-presentation, systemic involvement and clinical outcome. Eye (Lond).

[3] BE WL (1949). Sarcoidosis involving the orbit; report of two cases. Arch Ophthal.

[4] Prabhakaran VC, Saeed P, Esmaeli B (2007). Orbital and adnexal sarcoidosis. Arch Ophthalmol.

[5] René C (2006). Update on orbital anatomy. Eye (Lond).

[6] Simpson SM, Farmer J, Kratky V (2017). Acute orbital sarcoidosis with preceding fever and erythema nodosum. Can J Ophthalmol.

[7] Mavrikakis I, Rootman J (2007). Diverse clinical presentations of orbital sarcoid. Am J Ophthalmol.

[8] Demirci H, Christianson MD (2011). Orbital and adnexal involvement in sarcoidosis: analysis of clinical features and systemic disease in 30 cases. Am J Ophthalmol.

[9] Nasrazadani D, Quick AK, White WA (2017). An erosive lesion in the orbital apex as the presenting sign of sarcoidosis. Digit J Ophthalmol.

[10] Ryan TG, Curragh DS, Ellis D, Selva D, Davis G (2020). Orbital sarcoidosis with bony destruction. Clin Exp Ophthalmol.

[11] Park SS, Akella S, Kumthekar A, Barmettler A (2020). Extrapulmonary sarcoidosis in the orbit, brain, and heart. Am J Med.

[12] Shaikh ZA, Bakshi R, Greenberg SJ, Fine EJ, Shatla A, Lincoff NS (2000). Orbital involvement as the initial manifestation of sarcoidosis: magnetic resonance imaging findings. J Neuroimaging.

[13] Kellinghaus C, Schilling M, Lüdemann P (2004). Neurosarcoidosis: clinical experience and diagnostic pitfalls. Eur Neurol.

[14] Kim JS, Scawn RL, Lee BW, Lin JH, Korn BS, Kikkawa DO (2016). Masquerading orbital sarcoidosis with isolated extraocular muscle involvement. Open Ophthalmol J.

[15] Mukherjee B, Ambreen A, Alam MS (2017). Sarcoidosis of the ocular adnexa. Ann Allergy Asthma Immunol.

[16] Feizi P, Tandon M, Khan E, Subedi R, Prasad A, Chowdhary A, Sriwastava S (2021). Overcoming the elusiveness of neurosarcoidosis: learning from five complex cases. Neurol Int.

[17] Gaddam M, Ojinnaka U, Ahmed Z, Kannan A, Quadir H, Hakobyan K, Mostafa JA (2021). Sarcoidosis: various presentations, coexisting diseases and malignancies. Cureus.

[18] Maeng MM, Winn BJ (2019). Orbital infections and inflammations. The Columbia Guide to Basic Elements of Eye Care.

